# Does the Hyperglycemia Impact on COVID-19 Outcomes Depend upon the Presence of Diabetes?—An Observational Study

**DOI:** 10.3390/metabo12111116

**Published:** 2022-11-15

**Authors:** Inês Manique, Alexandra Abegão Matias, Bruno Bouça, Teresa Rego, Luísa Cortez, Teresa Sabino, António Panarra, Manfredi Rizzo, José Silva-Nunes

**Affiliations:** 1 Department of Endocrinology, Diabetes and Metabolism, Centro Hospitalar Universitário Lisboa Central, Hospital de Curry Cabral, 1050-099 Lisbon, Portugal; 2 Functional Unit of Internal Medicine 7.2, Centro Hospitalar Universitário Lisboa Central, Hospital de Curry Cabral, 1050-099 Lisbon, Portugal; 3 Department of Health Promotion, Mother and Child Care, Internal Medicine and Medical Specialties, School of Medicine, University of Palermo, 90100 Palermo, Italy; 4 Nova Medical School, Faculdade de Ciências Medicas, Universidade Nova de Lisboa, 1169-056 Lisbon, Portugal; 5 Health and Technology Research Center (H&TRC), Escola Superior de Tecnologia da Saúde de Lisboa, 1990-096 Lisbon, Portugal

**Keywords:** diabetes mellitus, hyperglycemia, COVID-19, clinical outcomes

## Abstract

Diabetes mellitus (DM) has emerged as a major risk factor for COVID-19 severity and SARS-CoV-2 infection can worsen glycemic control and may precipitate new-onset diabetes. At-admission hyperglycemia (AH) is a known predictor for worse outcomes in many diseases and seems to have a similar effect in COVID-19 patients. In this study, we aimed to assess the impact of AH regardless of pre-existing diabetes mellitus and new-onset diabetes diagnosis in the clinical severity of COVID-19 inpatients in the first months of the pandemic. A retrospective monocentric study on 374 COVID-19 inpatients (209 males) was developed to assess associations between AH (blood glucose levels in the Emergency Department or the first 24 h of hospitalization greater than 140 mg/dL) and severity outcomes (disease severity, respiratory support, admission to Intensive Care Unit (ICU) and mortality) in patients with and without diabetes. Considering diabetic patients with AH (N = 68;18.1%) there was a correlation with COVID-19 severity (*p* = 0.03), invasive mechanical ventilation (*p* = 0.008), and ICU admission (*p* = 0.026). No correlation was present with any severity outcomes in diabetic patients without AH (N = 33; 8.8%). All of the New-onset Diabetes patients (N = 15; 4%) had AH, and 12 had severe COVID-19; additionally, five patients were admitted to the ICU and three patients died. However, severity outcomes did not reach statistical correlation significance in this group. In nondiabetic patients with AH (N = 51; 13.6%), there was a statistically significant association with the need for oxygen therapy (*p* = 0.001), invasive mechanical ventilation (*p* = 0.01), and ICU admission (*p* = 0.03). Our results support data regarding the impact of AH on severity outcomes. It also suggests an effect of AH on the prognosis of COVID-19 inpatients, regardless of the presence of pre-existing diabetes or new-onset diabetes. We reinforce the importance to assess at admission glycemia in all patients admitted with COVID-19.

## 1. Introduction

The first cases of pneumonia caused by severe acute respiratory syndrome coronavirus type 2 (SARS-CoV-2) were identified in December 2019. After the rapid spread of this virus, on 11 March 2020, World Health Organization (WHO) declared COVID-19 a global pandemic [1]. Until now, according to WHO, there were more than 550 million confirmed cases and more than six million deaths. Although the scenario is quite different at the moment, in the first months of the pandemic more than one-fourth of hospitalized patients required Intensive Care Unit (ICU) admission [2]. Despite the development of vaccines against SARS-CoV-2, the pandemic is still raging and is challenginghealth services namely in Portugal. As a result, it is still critical to discover the key characteristics that influence a COVID-19 patient’s prognosis [3].

Several health conditions have been associated with a worse prognosis of COVID-19; diabetes mellitus had emerged as a major comorbidity for COVID-19 severity [4,5]. In one of the first original investigations that studied 138 hospitalized patients with COVID-19 in Wuhan, the prevalence of diabetes was 10% [6]. In the Wang W et al., study, regarding 2433 patients hospitalized in one hospital in Wuhan, diabetes was the second most common comorbidity, present in 14.3% of patients [7]. Diabetes prevalence is even higher when considering severe infection. Some studies reported that 22.2% to 27% of patients with severe COVID-19 present with diabetes [6,8,9].

SARS-CoV-2 infection may worsen glycemic control and may precipitate new-onset diabetes. The at-admission glycemia is a known predictor of critical illness and worse outcomes in many diseases; the same seems to apply to COVID-19 patients [3,10,11,12]. The prevalence of acute hyperglycemia in COVID-19 varies between studies. According to Li X et al., acute hyperglycemia occurs in about 22% of patients hospitalized for COVID-19, with 18.9% presenting diabetes [10]. However, other data registries showed that acute hyperglycemia occurred in about 50% of patients [13].

CORONADO study (Coronavirus SARS-CoV-2 and diabetes Outcomes) was a French nationwide multicenter observational study and one of the first to investigate the relationship between diabetes and COVID-19. They found an association between at-admission plasma glucose levels and the severity of COVID-19 [4].

Hyperglycemia occurs secondarily to the physiological response to stress hormones released during acute illness and also to metabolic dysregulations that impair insulin signaling [14]. Hyperglycemia can reduce the mobilization of leukocytes, decrease phagocytic activity, impair pulmonary endothelial function, and reduce antioxidant levels [15]. Hyperglycemia can also potentiate the harmful effects of cytokine storms in patients with COVID-19 pneumonia. There are some direct mechanisms described that explain the interaction between SARS-CoV-2 infection and glucose metabolism. The virus itself can induce pancreatic damage through angiotensin-converting enzyme 2 receptors, resulting in an impairment in insulin secretion [16,17]. Additionally, SARS-CoV-2 may enhance the pre-existing proinflammatory status common in diabetes, worsening the patient’s prognosis [16].

The present study was developed in a central Portuguese hospital dedicated to COVID-19, during the first months of the pandemic. In this study, we try to clarify the importance of hyperglycemia as a potential prognostic factor for COVID-19 inpatients, both with and without diabetes.

## 2. Materials and Methods

### 2.1. Study Design and Patients

We conducted a retrospective study on COVID-19 adult patients admitted to a medical ward (Internal Medicine Functional Unit 7.2) of Centro Hospitalar Universitário Lisboa Central (CHULC), a tertiary hospital, from 1 March 2020 to 31 October 2020. During the referred period, 374 patients were admitted to the ward.

All patients had been tested for SARS-CoV-2 by real-time polymerase chain reaction at admission in the emergency room. The medical care provided to each patient was selected for their clinical context, considering the clinical judgment and protocols provided by the Portuguese Directorate General of Health. This study has not influenced the type of care provided to the patients.

We extracted patients’ demographic and clinical data from electronic medical records: age, sex, gender, previous diabetes diagnosis, and blood glucose level at admission (obtained by collecting venous or arterial blood in the emergency department or in the first 24 h of admission to the ward), HbA1c measured during hospitalization, disease severity, and clinical outcomes. The disease severity was classified according to the national standards (Table 1).

The exclusion criteria in this study were: (1) Patients who were not confirmed by a positive result of SARS-CoV-2 detection in respiratory specimens by the reverse transcriptase polymerase chain reaction assay; (2) Patients with COVID-19 diagnosed only during hospital staying and not in the emergency room (3) Patients younger than 18 years old; (4) Patients without blood glucose measurement in the first 24 h after admission.

Considering these exclusion criteria and since we had no data from admission blood glucose in 14 patients (4 patients with previous DM and 10 patients without this diagnosis), only the 360 patients with admission blood glucose evaluated were considered in the present study.

The clinical outcomes analyzed included clinical severity, the need for oxygen therapy, Invasive Mechanical Ventilation (IMV), admission to the ICU, and mortality.

**Figure 1:**
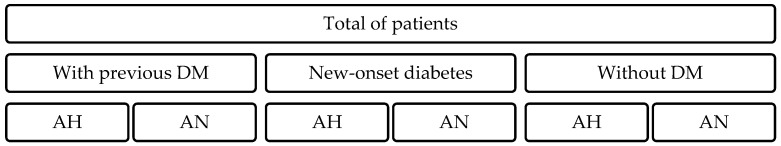
Groups considered for the analysis. Abbreviations: (AH, At-admission hyperglycemia; AN, At-admission normoglycemia; DM, Diabetes *mellitus*).

To analyze and compare clinical severity and outcomes, we considered three groups of patients (Figure 1): patients with previous diabetes, patients with new-onset diabetes, and patients without diabetes. In every group, we also considered two subgroups, patients with AH (blood glucose at-admission ≥ 140 mg/dL) and patients with at-admission normoglycemia (AN) (blood glucose at-admission < 140 mg/dL). For each group, each clinical severity outcome of COVID-19 was assessed and compared.

The Ethical Committee from Centro Hospitalar Universitário Lisboa Central (Comissão de Ética para a Saúde) approved this study (approval number 1270/2022) and waived patients’ Informed Consent due to the retrospective characteristics of the present study and anonymous data used.

### 2.2. Definitions

The case definitions of confirmed human infection with SARS-CoV-2 were under interim guidance from the WHO directives. We considered the presence of previous diabetes when the diagnosis of the disease and/or antidiabetic therapy was referred to in the patient’s medical record. New-onset diabetes was assumed in cases without a previous diagnosis and a measured HbA1c equal to or higher than 6.5% at any time during hospitalization. Absolute hyperglycemia at hospital admission was defined as any blood glucose level greater than 140 mg/dL, within the first 24 h after hospital admission or in the emergency department presentation, according to the American Diabetes Association guidelines and the joint recommendations of the Portuguese Society of Diabetology and the Portuguese Society of Internal Medicine on the management and treatment of hyperglycemia in hospital inpatients [18,19]. The stratification of the clinical severity of COVID-19 for each patient was based on the national clinical standards (Table 1).

### 2.3. Statistical Analysis

Descriptive statistics included means and standard deviations or median and interquartile range (IQR) for continuous variables (according to the presence/absence of a normal distribution) and frequencies and percentages for the categorical ones. Normal distribution was checked using the Shapiro-Wilk test or skewness and kurtosis. Categorical variables were compared with the χ2 method and continuous variables were compared using the Spearman correlation test. The association of AH with different outcomes was quantified as odds ratios (OR) that were adjusted using multiple logistic regression analyses with 95% Cis. Results with *p* < 0.05 (2-tailed) were considered results statistically significant. Data analysis was performed using IBM SPSS^®^ Statistics, version 23.

## 3. Results

During the period considered, 374 patients with COVID-19 were admitted to the ward, but admission blood glucose was evaluated in only 360 patients admitted. AH occurred in 119 patients (33.1%). In this subgroup of patients, 57.1% (n = 68) had diabetes mellitus previously diagnosed and 12.6% (n = 15) had new-onset diabetes with two of them presenting with diabetic ketoacidosis.

The demographic and clinical characteristics of each subgroup are shown in Table 2.

HbA1c was measured in 116 patients (32.2% of patients with also an at-admission glycemia measurement). The median HbA1c was 7.5% (IQR 6.2–9.3) in patients with diabetes and 5.9% (IQR 5.6–6.5%) in patients without diabetes (*p* < 0.001). In hyperglycemic patients with diabetes, the median HbA1c was 7.5% (IQR 6.2,9.3).

The characteristics of the analyzed groups in this study according to their glycemic status and their AH are depicted in Table 3.

From the total sample with at-admission glycemia data, 101 patients presented with a previous history of diabetes, 15 patients were diagnosed with new-onset diabetes, and 244 patients had no diabetes. The at-admission glycemia was higher than 140 mg/dL in 67.3% of patients with a previous diagnosis of diabetes (n = 68) and 15.8% (n = 39) of patients without the diagnosis. All of the fifteen patients with new-onset diabetes had hyperglycemia when admitted to the hospital. Admission blood glucose was higher in the groups of diabetic patients with at-admission hyperglycemia and the group with new-onset diabetes, with a median value of 211 mg/dL (IQR 168-269.8) and 203 (IQR 156–291) respectively.

In all groups, the majority of patients were Caucasian. Patients with new onset diabetes were younger, with a median age of 58 years old (IQR 43–78). At-admission blood glucose and HbA1c were higher in the group of diabetic patients with AH.

### 3.1. Clinical Outcomes

Clinical outcomes were analyzed according to the presence of AH or AN (Table 4) and according to diabetes diagnosis (Table 5).

#### 3.1.1. Total Population and Respective Glycemic Status at Admission

Compared with those without, patients presenting at-admission hyperglycemia were more likely to need oxygen therapy (*p* < 0.001), to be submitted to IMV (*p* < 0.001), to be admitted to the ICU (*p* = 0.002), or to present more severe presentations of the disease (*p* < 0.001). The mortality rate among patients was 18.3%, however, the presence of AH did not show a statistical correlation with this outcome.

#### 3.1.2. Clinical Outcomes in Each Group of Glycemic Status Patients and According to the Presence or Absence of At-Admission Hyperglycemia

The percentages presented in the table were inferred from de total value (n) of the respective column. In this table, we cannot include the patients without admission blood glucose measured (n = 14). *p*-values characterize the differences between the presence or absence of at-admission blood glucose in clinical outcomes.

The presence of AH was a predictive factor for the need for oxygen therapy in patients without diabetes (83.3% vs. 16.7%; *p* = 0.001). The likelihood for patients to be submitted to IMV was far greater in those presenting with AH in the group with a previous diabetes diagnosis as well as in those without diabetes (27.9% vs. 72.1%; *p* = 0.008 and 30.6% vs. 69.4%; *p* = 0.01, respectively). The same was observed for ICU admission with a higher probability of that need among patients presenting with AH in those two groups: 9.4% vs. 70.6% (*p* = 0.026) in the group with a previous diabetes diagnosis and 30.6% vs. 69.4% (*p* = 0.03) in those without diabetes. According to the presence or absence of AH, no significant difference was found in the risk of death in any of the groups considered.

We found AH to be a risk factor for higher severity of the disease among patients with a previous diagnosis of diabetes: critical disease in 30.9% of patients (n = 21) severe disease in 36.8% (n = 25), moderate disease in 9.6% (n = 6), mild disease in 14.7% (n = 10), and 8.8% (n = 6) being asymptomatic (*p* = 0.03).

**Figure 2:**
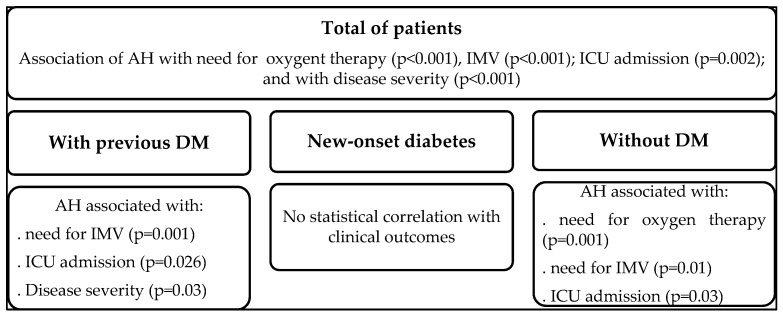
Summary of the results of our study (AH, At-admission hyperglycemia; AN, At-admission normoglycemia; DM, Diabetes *mellitus*; ICU, Intensive Care Unit; IMV, Invasive Mechanical Ventilation; n, number; NS—nonsignificant).

In Figure 2 we summarize the findings of our study.

We also performed multiple logistic regression analysis: indeed, after adjusting for age, sex, ethnicity and previous diabetes, AH was significantly and positively associated with the likelihood of oxygen therapy need (adjusted OR 3.2, 95% CI 1.81–5.68), invasive mechanical ventilation (adjusted OR 3.45, 95% CI 1.81–6.-57) and ICU admission (adjusted OR 2.77, 95% CI 1.51–5.10).

## 4. Discussion

The results of this study support the hypothesis that there is a direct impact of AH on the clinical outcomes of COVID-19, namely clinical severity, need for respiratory support (oxygen supply or IMV) or ICU admission. For most clinical outcomes, the impact of AH occurs regardless of a previous diagnosis of diabetes. In some cases, the impact of AH on clinical outcomes can be stronger than the presence of diabetes per se. Thus, our results support previous data regarding the impact of AH on clinical outcomes of SARS-CoV-2 infection.

Before this global pandemic, substantial evidence was already suggesting that patients with diabetes or AH could have worse outcomes in community-acquired pneumonia. Some recent studies showed similar results concerning COVID-19 [16,20]. However, the association between hyperglycemia and/or diabetes and the clinical outcomes of the infection seems complex.

Shi Q et al., found a higher proportion of ICU admission (17.6%) and more fatal cases (20.3%) of COVID-19 in patients with diabetes. [21] A retrospective cohort study showed a 59% higher risk for ICU admission and a 97% increased risk for mechanical ventilation among patients with diabetes [22].

In a large retrospective study, with COVID-19 inpatients from hospitals in Hubei province, it was observed that patients with diabetes needed more frequent oxygen therapy (76.9% versus 61.2%) and IMV (3.6% versus 0.7%) compared to those without diabetes. A higher mortality rate in patients with diabetes (7.8% versus 2.7%) was also observed [5]. However, Shi Q et al., did not find a statistically significant association between the presence of diabetes and in-hospital death in COVID-19 inpatients [21].

There is evidence also suggesting an increased risk for severe illness and mortality in critically and noncritically ill patients with COVID-19 who present with hyperglycemia (at admission or during hospitalization) [13,14,16,23,24,25].

A pooled analysis and meta-summary of literature described that SARS-CoV-2 infected patients who presented with raised blood glucose levels had an approximately threefold increased risk of mortality [16]. In a multicenter investigation with 495 critically ill patients, 35.8% of them with diabetes, it was found that hyperglycemia (≥140 mg/dL) was significantly associated with a prolonged stay in ICU, higher need for IMV, and increased risk for mortality [26]. A retrospective multicenter study of 2.041 COVID-19 patients hospitalized in China found AH (defined by the authors as glucose value ≥ 110 mg/dL) to be an independent risk factor for progression to critical disease and death in non-critically ill patients and an independent risk factor for in-hospital mortality in critically ill patients [26]. Mamtami M et al., also suggested that AH (value of the first 24 h after hospitalization ≥140 mg/dL) may be a good predictor for mortality [12].

Our study has also revealed that patients with AH had a higher risk of having a severe or critical clinical presentation compared with normoglycemic patients. The same pattern of comparison was verified for other clinical outcomes such as oxygen therapy (74.8% vs. 54.4%), IMV (28.6% vs. 11.6%) or ICU admission (30.3% vs. 14.9%) [16,20,27]. Contrary to other studies, we did not find older age to be a risk factor for AH either in patients with or in those without a previous diagnosis of diabetes [8,11,16].

We have observed that the presence of AH was a risk factor for worse outcomes in patients regardless of the presence or absence of a previous diagnosis of diabetes. However, opposite to other studies, we did not find those statistical associations in patients with new-onset diabetes maybe because of the sample size of this subgroup (15 patients) [28,29].

Our investigation revealed a higher proportion of patients with no previous diagnosis of diabetes but with AH who needed oxygen supply, IMV, and ICU admission. Concerning patients with a previous diagnosis of diabetes, AH showed to be a risk factor for the need for IMV, ICU admission, and a COVID-19 clinical severity. These findings suggest that stress hyperglycemia could play a role in the prognosis of patients hospitalized with COVID-19 regardless of the previous diagnosis of diabetes.

In addition to glycemia measured in the first 24 h after hospital admission, some studies investigated the association between other glycemic variables, such as fasting blood glucose, HbA1c or glycemic fluctuations during the hospital stay, with health outcomes related to COVID-19 [5,9,30,31,32,33,34].

Zhang et al., studied the outcomes of 166 COVID-19 inpatients in one hospital in Wuhan. After adjustment for confounding variables, the odds ratio for composite outcomes (ICU admission, use of either invasive or non-invasive mechanical ventilation, or death) was 5.47 (95% CI 1.56–19.82) for those with secondary hyperglycemia (patients with no history of diabetes, fasting glycemia ≥ 126 mg/dL and HbA1c < 6.5%) and 2.61 (95% CI 0.86–7.88) for those with diabetes (patients with fasting glycemia ≥ 126 mg/dL, previous history of diabetes or HbA1c ≥ 6.5%) [27].

A Chinese study with 548 COVID-19 inpatients, (18% with diabetes) suggested that patients who had higher mean levels of glucose during their first week of hospitalization were more likely to have a more prolonged hospital stay and greater risks for severe pneumonia, acute respiratory distress syndrome (ARDS) and death [33]. Similar results regarding the impact of AH and blood glucose control on COVID-19 severity were found by Fen X et al. [35]. Morse J et al., found mortality of 16.6% among cases with one or more glucose measurements in the hyperglycemic range (>180 mg/dL) during hospitalization, and this was associated with a substantial increase in the odds of mortality, regardless of a pre-existing DM diagnosis [36].

From a pathophysiologic perspective, the release of stress hormones is known to be the main component of the general adaptation to critical illness. However, stress hormone response results in persistent insulin resistance and hyperglycemia and this directly favors SARS-CoV-2 replication in human monocytes [15]. Accordingly, hyperglycemia and a history of diabetes are independent predictors of morbidity and mortality in patients with COVID-19 [37]. The hyperglycemic environment favors immune dysfunction through several pathways. Hyperglycemia decreases chemotaxis, reduces phagocytic activity, impairs endothelial function, induces apoptosis, and reduces antioxidant activity in the lungs promoting mitochondrial dysfunction [15,25]. Patients with diabetes and hyperglycemia have reduced natural killer cell activity, which may be a contributing factor to the susceptibility of these patients to more severe presentations of COVID-19 [37]. This proinflammatory milieu can be a contributing factor to the cytokine storm observed in COVID-19 patients, resulting in more severe illness and increased risk for sepsis [16,37]. Hypoxia is frequently accompanied by abnormal glucose metabolism at the cellular level [11]. Worsening in respiratory function is one of the major factors responsible for the detrimental effect of hyperglycemia in these patients [16].

Our study was conducted in one of the most important hospitals in Portugal managing COVID-19 inpatients during the peak of the pandemic. The results reinforce the strong association found between hyperglycemia and in-hospital worse outcomes in patients with COVID-19, regardless of the presence of previous diabetes. It also reinforces the importance to evaluate blood glucose levels in all patients and to strengthen blood glucose control in patients with diabetes to reduce the severity of SARS-CoV-2 infection [20]. The American Diabetes Association and the American Association of Clinical Endocrinologists recommend a target blood glucose range of 140–180 mg/dL for the majority of hospitalized noncritically patients [27,38]. Klonoff D et al., found that reaching a glucose value of ≤140 mg/dL or 141–180 mg/dL within 2 days after ICU admission or between 2 and 3 days in the non-ICU setting, respectively, is associated with reduced mortality [27]. Early correction of hyperglycemia in the course of COVID-19 could attenuate the release of inflammatory cytokines and reduce the virus’ ACE binding capacity. Taking these facts into account, we must pay attention to glucose control even in those patients without a previous diagnosis of diabetes.

Some studies have adopted a higher cut-off to consider hyperglycemia to study the impact on clinical outcomes in patients with diabetes. In our study, we used the same definition of AH in patients with and without diabetes, as suggested by ADA. Once we considered and evaluated only the effect of AH, we excluded the potential influence of steroid-induced hyperglycemia. Different criteria have been used to stratify COVID-19 clinical severity; in this study we considered national clinical standards to apply the classification. However, in other studies, different criteria do not seem to change the interpretation of results [31].

As shown in the Methods, we conducted the present retrospective study on all COVID-19 patients admitted to a medical ward between 1 March 2020 and 31 October 2020. This means that we included in our study only patients with COVID-19 who did not receive any vaccination. We believe that studying a wider group during a longer period of time was not the ideal approach for the aims of the present study, and other confounders would have been introduced, including the different schemes of vaccinations. Indeed, one of the strengths of the present study is the inclusion of patients during the first hit of SARS-CoV-2 infection, which can reveal better the impact of this coronavirus on clinical outcomes. We also need to mention that there was a delay in the preparation of the article and subsequent publication due to the pending approval by the local Ethics Committee.

There are some limitations that should be mentioned. First, a relatively small sample size possibly limits the statistical power of analyses, and it is possible that several observed trends would reach a statistical significance in a larger sample. Comorbidities such as hypertension, cardiovascular disease, chronic kidney, liver disease, and the presence of chronic diabetic complications were not taken into consideration for the results of our study. Since the vaccines were still not available in the referred period, we cannot exclude the impact of this potential confounder.

## 5. Conclusions

At-admission glycemia is an easy and inexpensive parameter to assess in all inpatients infected with SARS-CoV-2 [39]. It can be used as a prognostic marker in patients admitted to the hospital regardless of the presence of a prior diagnosis of diabetes [40,41]. Thus, the presence of AH can enable early and rapid identification of patients at risk for poor outcomes and improve risk stratification for COVID-19 inpatients [42,43]. Early glycemic control in patients with and without diabetes may be an effective therapeutic option to reduce poor outcomes in hyperglycemic COVID-19 inpatients. We also emphasize the need for a multidisciplinary approach in order to manage in the best effective way the potential acute complications as well as the chronic long-COVID sequels [44,45,46]. To ameliorate patients’ outcomes, large-scale multicenter studies are warranted to establish appropriate treatment guidelines for the management of hyperglycemia in COVID-19 inpatients.

## Figures and Tables

**Table 1:** Disease severity categories according to the Portuguese Directorate General of Health.

COVID-19 Disease Severity	Criteria
Mild disease	Mild symptoms without pneumonia or hypoxemia
Moderate disease	Pneumonia (fever, cough, dyspnea, tachypnea), peripheral oxygen saturation (SpO_2_) ≥90%, without hemodynamic instability
Severe disease	Pneumonia and at least one of the following: - tachypnea superior to 30 breaths per minute - breathing difficulty - SpO_2_ < 90% without supplemental oxygen - hemodynamic instability
Critical disease	Acute respiratory distress syndrome with PaO2/FiO2 <100 or septic shock

Adapt from: Directorate General of Health. “COVID-19: Abordagem do Doente com Suspeita ou Confirmação de COVID-19. Norma nº 004/2020 de 23 March 2020” actualized on 14 October 2020.

**Table 2:** Demographic and clinical variables of COVID-19 inpatients.

Population Variables	AN 241 (64.9%)	AH 119 (33.1%)	*p*
Glycaemia			<0.001
Median (IQR)	105 (91, 122)	191 (159, 227.5)	
Mean (SD)	111.7 (±22.2)	221.9 (±74.3)	
Sex			0.006
Female (%)	110 (45.6%)	50 (42%)	
Male (%)	131 (54.4%)	69 (58%)	
Age—years			0.62
Median (IQR)	71 (53, 86)	68.92 (55, 82)	
Mean (SD)	68.03 (±20.7)	72 (±15.7)	
Minimum	21	29	
Maximum	100	96	
Female median age (IQR)	75.5 (56, 86.3)	74 (61.7, 86)	
Male median age (IQR)	70 (50, 86)	70 (52, 77.5)	
Origin			0.03
Caucasian (%)	215 (89.2%)	96 (80.7%)	
Not Caucasian (%)	26 (10.8%)	23 (19.3%)	
Previous DM diagnosis			<0.001
Without previous DM (%)	208 (86.3%)	51 (42.9%)	
(New-onset diabetes)		15 (12.6%)	
With previous DM (%)	33 (13.7%)	68 (57.1%)	
HbA1c			<0.001
Median (IQR)	5.9% (5.6, 6.4)	7.5% (6.2, 9.3)	

Abbreviations: AH, At-admission hyperglycemia; AN, At-admission normoglycemia; DM, Diabetes Mellitus; IQR, interquartile range; n, number; SD, standard deviation.

**Table 3:** Demographic and clinical variables of COVID-19 inpatients according to their glycemic status.

	Previous DM (28.1%; n = 101)		*p*	New-Onset Diabetes (4.2%; n = 15)		*p*	No DM (67.8%; n = 244)		*p*
	AH 67.3% (n = 68)	AN 32.7% (n = 33)		AH 100% (n = 15)	AN 0% (n = 0)		AH 14.8% (n = 36)	AN 85.2% (n = 208)	
Sex			0.68			-			0.44
Female (%)	30 (44.1%)	16 (48.5%)		7 (46.7%)	-		13 (36.1%)	94 (45.2%)	
Male (%)	38 (55.9%)	17 (51.5%)		8 (53.3%)	-		23 (63.9%)	114 (54.8%)	
Age—years			0.37			-			0.66
Median (IQR)	72 (52.75–85)	78 (58.5–86)		58 (43–78)	-		74 (58–82)	71 (51, 85.75)	
Mean (SD)	69.47 (±14.6)	72.06 (±15.9)		59.3 (±19.1)	-		69.7 (±15.8)	67.39 (±21.3)	
Minimum	42	38		33	-		29	21	
Maximum	96	100		90	-		94	100	
Ethnicity			0.52			-			0.13
Caucasian (%)	54 (79.4%)	28 (84.8%)		11 (73.3%)	-		31 (86.91%)	187 (89.9%)	
Non-Caucasian (%)	14 (20.6%)	5 (15.2%)		4 (26.7%)	-		5 (13.9%)	21 (10.1%)	
HbA1c			<0.001			-			<0.001
Median (IQR)	7.9 (6.4–9.0)	6.5 (5.7–7.2)		7.6 (6.8–11.1)	-		5.7 (5.2–6.2)	-	
At-admission glycemia (mg/dL)			<0.001			-			<0.001
Median (IQR)									
Mean (SD)	211 (168–269.8)	118 (105.5–132)		203 (156–291)	-		176 (154.5–200.5)	109 (94–125)	
	224.6 (±71.9)	118.6 (±20.6)		224.9 (±107.5)	-		182.7 (±33.6)	108.8 (±22.5)	

Abbreviations: AH, At-admission hyperglycemia; AN, At-admission normoglycemia; IQR, interquartile range; n, number; SD, standard deviation.

**Table 4:** Clinical outcomes according to at-admission glycemia.

	Total (n = 360)	At-Admission Glycemia Was Measured (n = 360)		
		AN (n = 241)	AH (n = 119)	*p*
Oxygen therapy				<0.001
Not done (%)	140 (38.9%)	110 (45.6%)	30 (25.2%)	
Done (%)	220 (61.1%)	131 (54.4%)	89 (74.8%)	
IMV				<0.001
Not done (%)	298 (82.7%)	213 (88.4%)	85 (71.4%)	
Done (%)	62 (17.2%)	28 (11.6%)	34 (28.6%)	
ICU admission				0.002
Not admitted (%)	288 (80%)	205 (85.1%)	83 (69.7%)	
Admitted (%)	72 (20%)	36 (14.9%)	36 (30.3%)	
Mortality				0.692
Survivor (%)	294 (81.7%)	199 (82.6%)	95 (79.8%)	
Non-survivor (%)	66 (18.3%)	42 (17.4%)	24 (20.2%)	
COVID-19 severity				<0.001
Asymptomatic, n (%)	46 (12.8%)	36 (14.9%)	10 (8.4%)	
Mild, n (%)	69 (19.2%)	54 (22.4%)	15 (12.6%)	
Moderate, n (%)	34 (9.4%)	27 (11.2%)	7 (5.9%)	
Severe, n (%)	145 (40.3%)	93 (38.6%)	52 (43.7%)	
Critical, n (%)	66 (18.3%)	31 (12.9%)	35 (29.4%)	

Abbreviations: AH, At admission hyperglycemia; AN, At admission normoglycemia; ICU, Intensive Care Unit; IQR, Interquartile range; IMV, Invasive Mechanical Ventilation; n, number; NS—non-significant; SD, Standard Deviation. The percentages presented considered the n value of the respective column.

**Table 5:** Clinical outcomes considering the glycemic status and diabetes diagnosis.

Variables	Previous DM (28.1%; n = 101)				New-Onset Diabetes (4.2%; n = 15)			No Previous DM (67.8%; n = 244)			
	AH (n = 68)	*p*	AN (n = 33)	*p*	AH (n = 15)	AN (n = 0)	*p*	AH (n = 36)	*p*	AN (n = 208)	*p*
Oxygen therapy		0.099		0.282		-	0.077		0.001		0.255
Not done (%)	21 (30.9%)		16 (48.5%)		3 (20%)			6 (16.7%)		94 (45.2%)	
Done (%)	47 (69.1%)		17 (51.5%)		12 (80%)			30 (83.3%)		114 (54.8%)	
IMV		0.008		0.079		-	0.092		0.01		0.175
Not done (%)	49 (72.1%)		31 (93.9%)		11 (73.3%)			25 (69.4%)		182 (87.5%)	
Done (%)	19 (27.9%)		2 (6.1%)		4 (26.7%)			11 (30.6%)		26 (12.5%)	
ICU admission		0.026		0.108		-	0.067		0.03		0.184
Not admitted (%)	48 (70.6%)		30 (90.9%)		10 (66.7%)			25 (69.4%)		175 (84.1%)	
Admitted (%)	20 (29.4%)		3 (9.1%)		5 (33.3%)			11 (30.6%)		33 (15.9%)	
Mortality		0.647		0.644		-	0.477		0.141		0.845
Survivor (%)	57 (83.8%)		28 (84.8%)		12 (80%)			26 (72.2%)		171 (82.2%)	
Not survivor (%)	11 (16.2%)		5 (15.2%)		3 (20%)			10 (27.8%)		37 (17.8%)	
COVID-19 severity		0.03		0.312		-	0.321		0.06		0.197
Asymptomatic, n (%)	6 (8.8%)		5 (15.2%)		1 (6.7%)			3 (8.3%)		31 (14.9%)	
Mild, n (%)	10 (14.7%)		9 (27.3%)		1 (6.7%)			4 (11.1%)		45 (21.6%)	
Moderate, n (%)	6 (9.6%)		2 (6.1%)		1 (6.7%)			0 (0%)		25 (12%)	
Severe, n (%)	25 (36.8%)		15 (45.5%)		8 (53.3%)			19 (52.8%)		78 (37.5%)	
Critical, n (%)	21 (30.9%)		2 (6.1%)		4 (26.7%)			10 (27.8%)		29 (13.9%)	

Abbreviations: AH, At-admission hyperglycemia; AN, At-admission normoglycemia; DM, Diabetes mellitus; ICU, Intensive Care Unit; IMV, Invasive Mechanical Ventilation; n, number.

## Data Availability

The data are not publicly available due to privacy restrictions.

## Abbreviations

AH—At-admission hyperglycemia; AN—At-admission normoglycemia; CHULC—Centro Hospitalar Universitário Lisboa Central; CI—Confidence interval; DM—Diabetes mellitus; ICU—Intensive Care Unit; IMV—Invasive Mechanical Ventilation; IQR—Interquartil range; OR—Odds ratio; SARS-CoV-2—Severe Acute Respiratory Syndrome Coronavirus-2; SD—Standard deviation; WHO—World Health Organization.
